# From defensive reasoning to innovation: how digital tools foster positive emotions in organizations

**DOI:** 10.1186/s40359-025-02486-6

**Published:** 2025-02-20

**Authors:** Guyang Tian, Yang Yang, Xue Zhang, Manlu Zhao, Yezhuang Tian

**Affiliations:** 1 Harbin Institute of Technology, Harbin, China; 2 Shanghai Normal University, Shanghai, China

**Keywords:** Defensive reasoning, The broaden-and-build theory of positive emotions, Digital management tools, Narrative networks analysis, Cultivate innovation

## Abstract

Researchers of organizational learning and innovation have frequently claimed that defensive reasoning is an inducer of negative emotions present in most organizations, inhibiting continuing innovation and development and individuals’ effective learning. Although a significant amount of research has been conducted on the factors that reduce defensive reasoning in an organization, the breakthrough process and its outcomes have not yet been studied. This study uses narrative network analysis on longitudinal data from a Chinese tourist resort to explore how organizational factors—such as institutional environments, social media platforms, and virtual-reality role transformations—help employees overcome defensive reasoning and foster innovation. It demonstrates how digital management tools and corporate social platforms transform negative emotions into positive, creativity-enhancing actions. By analyzing individual defenses from both general and job position perspectives, the study introduces a comprehensive theoretical framework that highlights the role of emotional guidance and digital tools in reducing defensiveness, promoting collaboration, and improving organizational effectiveness and innovation.

**Supplementary Information:**

The online version contains supplementary material available at 10.1186/s40359-025-02486-6.

## Introduction

As the market environment has become more competitive, organizational innovation has become the central issue and a significant source of competitive advantage for modern businesses [1]. Today’s innovations require external expertise. Enterprises should increase learning and innovation to sustain innovation [2]. Defensive reasoning is a major obstacle to organizational learning and innovation during enterprise development [3]. Although digital technologies provide new conditions for innovation, organizational defensive behaviors often emerge alongside technological advancements. Particularly when faced with change, individuals and organizations may resort to defensive mechanisms to resist the adoption of new technologies, thereby affecting organizational adaptability and innovation capacity [4]. Defensive reasoning is an integral self-protection strategy in which people adopt defensive behaviors such as avoidance, silence, and knowledge concealment to avoid embarrassment or conflict [5]. Digital technologies struggle to detect deeply ingrained defensive mechanisms within individuals, which, in rapidly changing environments, are likely to be repeatedly overlooked and may even exacerbate information distortion. Some scholars note that a great risk for organizations in knowledge dissemination comes from believing that false and defensive information is more powerful than true information [6]. Defensive reasoning exists in most organizations, saying that it directs individuals to subconsciously self-censor specific ideas, making it very challenging to identify [7].

Scholars have established that negativity is a prerequisite for inducing defensiveness [8, 9] and can have a strong destructive effect on organizations [10]. Organizational defensive thinking restricts innovation, which in turn exacerbates negative emotions in work. Although scholars have long sought to propose solutions based on defensive characteristics. In particular, organizations take actions such as strengthening organizational feedback links [11], altering leadership styles [12], encouraging open thinking and communication between organization members, reflecting on one’s behavior [13], and building an inclusive team climate that is open to learning from mistakes [14]. Little research has been conducted on the mechanism of emotions in overcoming defensive reasoning at the individual level. Organizational defensive reasoning entails both individual and social interaction [3, 15], which requires a comprehensive breakthrough process. In addition, the strategy proposed in previous studies is aimed at a general perspective, only a few studies focused on the defensive behavior of managers [16]. It is therefore necessary to increase attention to the defensive phenomena from the perspectives of both leaders and subordinates. Because the affective responses of employees to the behavior of leaders vary based on workplace interaction [17]. Defensive behaviors exhibited by employees may stem from their evaluation and interpretation of the leader or may represent mimicry of the leader’s actions. Leaders are perceived as key sources of attribution for their subordinates’ interpretations of daily behaviors and thoughts [18], and their cognition, behaviors, motivations, and the manner in which they distribute cognitive power significantly impact subordinates. These dynamics, in turn, influence team atmosphere and performance [19]. In conclusion, it is essential to have a comprehensive process that not only encompasses the interaction between leaders and employees but also separates their perspectives for discussion.

With the advancement of digital transformation, the means of enterprise management are continuously upgrading. The widespread use of digital management tools enables employees to communicate more conveniently, reducing the potential awkwardness that may arise from face-to-face interactions and thereby alleviating employees’ defensive pressures. In the digital background, organizational structures are trending towards borderless, flat, and decentralized structures [20, 21]. This flattening structure makes organizational boundaries more flexible, allowing employees to autonomously adjust to changes in tasks and objectives, free from the constraints of traditional organizational structures, and stimulating a series of positive emotions such as autonomy, sense of control, and achievement among employees. According to the broaden-and-build theory of positive emotions, positive emotions may help individuals avoid shame or cope with fearful situations, promoting work achievement and social interactions [22]. In digital management platforms, different individuals form dense virtual communities through interactions based on common goals, with identities among employees fluctuating with the emergence of virtual communities. In virtual platforms, employees can drop their guards, communicate their true thoughts, share knowledge, and thus foster an open innovation network [23]. The use of digital tools provides employees with functionalities for information exchange and emotional expression, making them effective instruments for organizations to overcome individual defensive reasoning. However, the mechanisms through which digital tools mitigate individual defensiveness remain unclear. Additionally, it is essential to explore how individuals who embrace openness interact on digital platforms.

Scholars advocated developing a framework based on empirical research of multi-layer and multi-participant network dynamics, That, narrative network analysis is a method that visualizes the action patterns in an organization through different sets of actors and includes insights into single, isolated events and the process toward a holistic perspective [24]. Existing narrative network diagrams depict a comprehensive process, but it is difficult for researchers to promptly identify the development context in the face of highly complex development events. Consequently, the narrative network analysis diagram incorporates focal event development factors to facilitate comprehension. Based on this, we present a theoretical discussion on the holistic view of “network processes” as a relationship between individual and organizational institutions to overcome defensive reasoning and develop a network-process meta-framework to complement framework [25, 26].

This research draws on a narrative network analysis of longitudinal case data (2018–2019) collected from a tourist resort in China, analyzing focal events that occurred over a continuous period within the enterprise. We make three contributions to the literature: First, we demonstrate the comprehensive process through which employees, who initially engage in defensive reasoning, gradually interact and open up their hearts under the influence of management tools and social platforms, constructing positive emotions step by step. Second, we track the timeline of longitudinal cases and divide the characteristics and breakthrough process of individual defense into the general individual perspective and the job position perspective, enriching the perspectives and conclusions of previous related studies. Finally, by combining the theory and practical methods of breaking through individual defensive reasoning with different management tools in the digital context, we can transform negative emotions triggered by individual defense from multiple perspectives, stimulate and disseminate positive emotions, thereby helping organizations improve competitive efficiency and achieve innovation goals.

## Literature review

### Defensive reasoning

Our definition of defensive reasoning is derived from the conceptual definition proposed by Argyris (1990) actions or policies that prevent individuals or segments of the organization from experiencing embarrassment or threat. Argyris believes self-assessment—whether open ideas embarrass or expose—motivates defensive action [7]. Private self-assessment leads to deliberate reflection on behavior [27]. Defensive reasoning involves ambiguity and a refusal to be honest. These behaviors originate from the habits formed by people’s subtle behaviors in the social environment and make people involuntarily use defensive reasoning to think and act in daily activities. Argyris observed that defensive reasoning is a very common and unpredictable behavior [7]. Simultaneously, defensive reasoning involves not only individual behavior but also social interaction [3, 15]. People may unconsciously reinforce each other’s defensive behaviors through social activities, and this reciprocal defensive behavior leads people to believe that they (or others they care about) will be reprimanded for revealing their true thoughts [28]. In a given situation, individuals exhibit defensive behavior because they believe it is reasonable to avoid embarrassment and threats, and they are influenced by a situational culture that sees failure and conflict as embarrassing [29].

Defensive reasoning, which protects individuals from threats and threat-related problems [27], is regarded as part of a self-protection mechanism which is one of the quickest solutions to problems with unverified or missing information [5]. This behavior will lead people to retain, distort, or even delete knowledge to a certain extent, resulting in concealment or misrepresentation, which has an important impact on organizational success and even survival [30, 31].

The existing related research makes suggestions from different aspects. Many scholars have suggested that organizations can reduce the generation of defensive reasoning by encouraging employees to conduct knowledge inquiry [32]; promoting open dialogue and improving communication skills [33]; building a safe team atmosphere [14]; and introducing external coaching to help the enterprise identify defensive reasoning [34]. Leaders who are willing to empower employees with control over available information informed choices, and an understanding of personal responsibility for monitoring their performance will also reduce defensive reasoning and promote learning behaviors within the organization [35]. To reduce the generation of defensive reasoning, people need to think openly communicate with all concerned, and consciously reflect on their behavior [13].

### The broaden-and-build theory

The broaden-and-build theory of positive emotions was first proposed by Fredrickson (2001) [36]. Emotion is a strong feeling of a human being, it points to the individual’s psychological reaction. Before the theory was developed, negative emotions (such as anger, fear, disgust, and guilt) were heavily studied by psychologists. He argued that although specific behavioral tendencies can explain most of the manifestations and functional mechanisms of negative emotions, they cannot be applied to positive emotions, because positive emotions do not seem to provide coping with life-threatening situations, and usually do not point to a specific behavioral tendency with positive, positive emotions should have different adaptive meanings than negative emotions [36].

The broaden-and-build theory of positive emotions states that positive emotions can expand the range of an individual’s instantaneous thinking behavior, and then build lasting personal resources (intellectual, physical, psychological, and social), thus bringing long-term adaptability to the individual’s benefit [36]. The theory has two core assumptions, the broaden hypothesis and the build hypothesis.

The broaden hypothesis holds that positive emotions, relative to negative emotions and neutral states, widen the array of thoughts, action urges, and percepts that spontaneously come to mind. When faced with difficulties, positive emotions can better match task switching than negative emotions [37]. The social-cognitive role of positive emotions in teamwork has also been verified by many scholars. For example, positive emotions will expand the circle of trust among members and allow them to form a common group identity, so they are more likely to regard “them” as “us” [38].

The broaden-and-build theory of positive emotions has been widely applied across various fields to explain how positive affect expands cognitive and behavioral capacities while building long-term personal and social resources. In organizational psychology, it enhances employee creativity, cognitive flexibility, and perspective-taking, particularly in dynamic service industries like hospitality [39]. In sales and marketing, it links subjective well-being to adaptive selling and sales creativity, mediated by coping strategies and moderated by organizational identity [40]. Therapeutically, the theory underpins interventions like the Flash Technique, aiding trauma processing and emotional resilience through positive emotions [41]. It has also been applied to stress management, where art-based interventions reduce stress and promote well-being among college students [42]. In the context of sports fandom, the theory explains how nostalgia fosters subjective well-being, social connectedness, and behaviors like travel intentions [43]. Foundational research across these fields demonstrates its role in enhancing creativity, receptivity to new information, and adaptive responses, showcasing its broad utility in addressing psychological, social, and organizational challenges [44].

The build hypothesis argues that the expanded instantaneous thought–action range can help individuals build lasting personal resources, bringing long-term benefits to the individual. The function of the expansive form of positive emotions is to spur the development of resources, placing people on positive growth trajectories. Those more willing to express positive emotions appear to be more resilient and resourceful and perform at their best [45].

### Digital transformation and organizational emotions

With the continuous advancement of digital technologies, Digital Human Resource Management has become an essential component of corporate strategy and operations. Digitalization refers to the way digital technologies are leveraged to transform existing business processes, emphasizing the role of digital technology interventions in shaping new organizational structures [46]. This process involves not only a technological upgrade but also the reconfiguration of entire business operations. In this evolving landscape, emotional intelligence (EI) has gained considerable attention, particularly in how it interacts with organizational changes brought about by digital transformation. Emotional intelligence is defined as the ability to monitor one’s own and others’ emotions and feelings and to use this information to guide thinking and actions [47]. It is a key differentiator between high-performing individuals and their average counterparts. Further studies underscore the role of EI across various sectors. For instance, in the hospitality industry, EI has been shown to significantly affect both job satisfaction and employee performance. Employees with higher emotional regulation and social skills tend to report greater job satisfaction and demonstrate higher performance levels [48]. These findings highlight the importance of EI in industries where interpersonal interactions are critical to success, emphasizing the need for organizational leaders to foster and cultivate emotional intelligence within their workforce.

Similarly, the integration of digital tools in sectors such as banking is transforming not only technological structures but also how organizations manage uncertainty. Ntasis, Kountzakis [49] suggests that digital transformation in the financial sector is not only about risk management but also about addressing the emotional and cognitive responses to uncertainty, which are shaped by both digital tools and the human capital interacting with them. Moreover, Koronios, and Dimitropoulos [50] examine the relationship between employees’ emotional intelligence (EI) and ethical values, and their impact on motivation and performance in small and medium-sized enterprises (SMEs). Their findings indicate that strong ethical values and high EI enhance employee motivation, which in turn boosts organizational performance. This highlights the importance of these traits for managerial strategies aimed at improving employee engagement and productivity.

As organizations embrace digital transformation, it is crucial to consider the emotional impact of digital tools. Strategically leveraging technology can enhance positive emotions, reduce defensive behaviors, and improve overall productivity. Recognizing the role of EI in digital strategies will help organizations foster emotional well-being while navigating the challenges associated with digital change.

## Method

### Narrative network analysis

Narrative is an activity that takes place in a group of narrators, each of which is a speaker and a listener [51]. Narratives do not represent a single shared understanding of a field; rather they represent a team’s diverse views of events and actions. A narrative can describe an entire life story or an organization’s history or be a description of a specific event or process [52]. Narrative networks are a method for expressing patterns of action that retain possibilities and alternatives [25]. Compared with traditional process models and workflow diagrams, it can highlight the flexibility and fluidity of the day-to-day formulation process and can present multiple perspectives to form a holistic view for understanding the processes unfolding in an organization [53]. Narrative network analysis is a useful tool that has been rapidly expanded and applied by drawing on and borrowing narrative analysis methods from the social sciences [54].

Ricoeur [55] argued that, through the narrative process, individuals not only shape their identity and relationships with others but also allow us to understand their relationship to their surroundings. As the trajectory of events unfolds, the contrasts and connections formed between narrative segments help researchers understand the “non-locality” of events, in which events in one space-time location are immediately related to events in another location. Through narrative network analysis, researchers can understand how certain policy-relevant narratives surface, persist and change in time and space, weaving together actors in the network [26]. According to the narrative network approach, researchers analyze the narrative network by separating the narrative network into different narrative segments and presenting actions across time, which helps us to solve the hidden relationship produced by different individuals.

The concept of narrative networks is similar to what [56] called action networks. The narrative network provides a theoretical explanation of how enacting different steps influences the possible paths in a process. The nodes describe the connection from one activity to another. A path describes a process of action that has been completed or may be performed, and it is seen as a series of steps formulated over time [57].

Narrations of network processes depict focal and contextual events (what happened, how it happened), actors (who was involved), context (where it happened when it happened, in which settings), proposals for the explanation, and process triggers (why it happened), and attitudes toward the process. Researchers can understand how the network processes evolve by observing the relevant constructs in the activity patterns in the focal events and the interactions between the structures [58].

According to Pentland and Feldman (2008), the method of constructing narrative network analysis, the narrative network needs to include actors and actions, narrative fragments, and narratives. Actors are defined as participants in a narrative network; they make up the smallest part of the narrative network, like atoms or elements. Actions connect actors through common objectives. Narrative fragments represent basic nodes of the focal event in a narrative network, consisting of at least two behavioral actors and some behavior that occurs between them. The focal event generally refers to a subpart of the network process that is the primary focus of the analysis, thus is comparable to the activity concept to some extent. A narrative represents a coherent process that connects a coherent progression or sequence of events with a purpose or goal and has a distinct beginning, middle, and end. Narrative network diagrams represent patterns of action in an organization and consist of several interconnected nodes.

Narrative Network Analysis has been applied across diverse fields to explore the dynamic interplay between actions, structures, and meaning-making processes. For example, Pentland and Feldman [59]introduced narrative network analysis to represent patterns of technology use in organizations, highlighting how modularity and recombinability in information and communication technologies align with evolving organizational forms. This approach enables insights into design processes and organizational routines. In Constantinides and Barrett [60]’s study, narrative network analysis was used to analyze coordination practices during emergency responses in Greece, emphasizing the temporal and situated nature of coordination through narrative performance. The approach has also informed debates on personal identity, with Sauchelli [61]’s study exploring how narrative perspectives challenge psychological theories underpinning life-extending enhancements. Further, Decuypere [62]’s study demonstrated how narrative network analysis can trace dynamic patterns in organizational routines by mapping action paths, offering mechanisms to explain changes over time, as seen in a video game development project. In Wibisono, Sammon [63]’s study, researchers modeled workaround processes in organizations using narrative network analysis, providing a method to unpack data-centric issues such as privacy and loss, while fostering managerial awareness of workaround practices. These examples demonstrate narrative network analysis’s versatility in examining complex, contextualized systems across organizational, technological, and social domains.

### Data collection and case description

The case of this study was selected from a large integrated tourism resort in China. From 2016 to 2021, a total of seven field investigations were conducted to continue the case company follow-up. The selected focal events for this investigation occurred between April 2018 and June 2019. Interviews, observations, documents from the enterprise’s social media, and internal assessment data for 14 months (2018–2019) with supervisors and administrators of varying levels and front-line employees provided the data for this study. Table 1 lists the data sources used in this investigation.

**Table 1:** Summary of data sources

Type	Data source	Words (thousands)	Period
Filed observation	The research team—seven times		2016–2021
Semi-structured interview	General manager, deputy general manager, supervisor, and employees	193	2018–2019
Documents in the enterprise’s social media	Communication in discussion posts	110	2018–2019
Enterprise’s internal assessment data	Weekly, monthly, quarterly, and annual assessment data	264	2018–2019

The case enterprise in this study is a large-scale tourist ski resort with more than 1,300 employees in Northeast China. General Manager MCY has leveraged various digital management tools to overcome layers of bureaucracy, improve organizational efficiency, and cultivate employees’ ability to actively identify problems. Based on the field observations and interviews with General Manager MCY, the company faced challenges related to individual defensive reasoning, such as high-level leaders being overly focused on controlling others and often unwilling to listen to diverse perspectives, while lower-level employees tended to exhibit sluggish behavior and superficial compliance. After extensive exploration, General Manager MCY adopted an open authorization system model, leveraging electronic performance evaluations and internal corporate social media platforms to help employees release negative emotions and shed individual defenses.

The electronic performance evaluation includes the Debriefing Process and the Competition Process^1^. These digital management tools differ from traditional management tools in the following aspects:

1. A networked hierarchical structure—for example, Department B is allowed to participate in the decision-making of the management affairs of Department A;
2. Open and transparent information, employees and supervisors using their actual names on corporate social-media platforms, also their evaluation data is accessible on any level;
3. Transparent procedural fairness and rules, displayed by the fact that members’ promotions and demotions are governed by management rules rather than the preferences of the leaders;
4. Positive error management, which is exemplified by the fact that, when errors occur, employees can accept criticism without prejudice and are willing to correct errors because the company has an inclusive culture;
5. Dissipation (A way that needs to lose its original internal energy to gain new energy, dissipation in the context of this study represents candid sharing of ideas and previous practices with others) and openness, embodied in the point reward system to motivate employees to participate in management decision-making by asking questions and making suggestions.

This research selects the focus events in the two stages of the enterprise development to illustrate: Stage 1: in 2018, employees’ defensive reasoning showed that they avoided being frank and confrontational. Stage 2: in 2019, long-term leadership by a single leader created a closed and defensive structure in an initially effective team. The managers’ biased approach created inequity between team “insiders” and “outsiders.” This caused tension to emerge in employees’ relationships, making them hesitant to express their genuine feelings.

Case on stage 2 follow-up after nearly four months showed the enterprise members overcame the defensive reasoning, developed their potential in the subsequent management work, and made many innovative achievements.

## Data analysis

We analyze using the narrative network analysis method. We regard the narratives of various events collected from the internal communication platform of the enterprise as a narrative network, with the beginning and the end of an event as the boundary. This narrative boundary facilitates the comparison and interpretation of organizational members’ communication states when engaging in efforts to dismantle internal defenses. It also provides a framework for explaining mechanisms that effectively overcome defensive reasoning. Additionally, through narrative network mapping, the overall network process of overcoming defensive reasoning can be visually represented.

To frame our analysis, we construct narrative networks based on concepts proposed by Pentland and Feldman (2008), namely, actors and actions, narrative fragments, and narratives. Table 2 summarizes the company’s related concepts and examples. We will discuss more details of each action node in the following analysis.

**Table 2:** Summary of related concepts and examples

Concept	Description	Focal event and exemplar data
Actors and actions	Actors: staff, supervisor, deputy general manager, general manager	**Attack on the defense**: emotional motivation field—a Debriefing Process for collaborative encouragement The Debriefing Process has been completed. Compared with the past, the biggest highlight of this debriefing is that the debriefing is carried out with the team as a whole, with full participation, creating a self-organizing environment and achieving the initial self-organizing effect. (Supervisor ZSJ) **Breakthrough condition 1**: The soil of positive emotions—institutional environment Thanks to the open environment of the company, I can feel my value more, and I will work harder in the future. (Staff member ZBY) **Breakthrough condition 2**: Positive emotions drive —virtual-reality multi-role transformation **Breakthrough condition 3**: Positive emotion communicator—enterprise social-media platform We must carry forward our positive energy and resist our negative energy. (Staff member XKC) **The disintegration of defense**: emotional dissipation field—reflection during the competition process Event 1: The structure of defensive team culture leads to successive failures. The team’s results, led by the insiders who rely on parental control, are not good. I will conduct an in-depth reflection. (Supervisor PZW) Event 2: team reflection gains another opportunity. The failure of this competition did not knock down our entire team. We have formed a team cohesion from a mess of sand to the present, inseparable from everyone’s efforts. (Staff member XXR) **Defense breakthrough**: The effect of positive emotions—members innovate independently, making a new marketing record After a month of strategies, we finally got off to a good start. The whole department analyzed from a strategic point of view to all-round contact with the principals and then to the independent promotion of all members. Everyone’s unremitting efforts have created a new theme of park marketing. (Deputy General Manager ZZM)
	Actions: actions connect actors through common goals	
Narrative fragment	An essential node in a narrative network consisting of at least two actors and some action taking place in or between them	**Breakthrough individual defensive reasoning** Today’s sharing aims to create an open environment; once everything has been said, instead of complaining, everyone should provide genuine feedback based on the evaluation. (Deputy General Manager WXX) **Encourage positive emotions** People’s potential is unlimited; the key is whether there is an environment to develop their potential. The environment creates people, and people are the main body changing the climate. I believe that everyone will cherish the environment and use continuous actions to create a greater development environment. (General Manager MCY)
Narrative	A coherent progression or sequence of events with a purpose or goal and a distinct beginning, middle, and end	April 2018: attack to defensive reasoning March 2019: overcoming defensive reasoning June 2019: members innovate independently

### Actors and actions

The main actors in the case include staff members WK, SGH, ZSJ, and LMH; supervisors CYY, LXL, and PZW; deputy general managers WXX and ZZM; and general manager MCY. The main roles assumed by these actors in breaking through defensive reasoning at the individual level, Table 3 lists the roles and key actions of each actor and their views and perspectives on the focal events.

**Table 3:** Actor roles and key actions

Actor	Role	Key action(s)	Examples
Staff member WK	Bench-marker	Share successful experiences	“Our organization has such a good mechanism and even more good leaders. If we don’t work hard, we will be eliminated.”
		Encourage others	“Combining theory with practice is the real learning attitude. Although we started late, the emphasis is on participation.”
Staff member SGH	Exemplary	Promote positive emotions	“Use our changes to stimulate every member in the module. Only by win-win can we win the future.”
		Participate in discussions actively	“Focus on participation rather than wait and see, and need to be present to absorb knowledge. If you don’t do it, you’ll never know what’s wrong, and we get reviews.”
		Help others analyze problems and encourage action	“Rome wasn’t built in a day. If you can’t change the environment, you have to change yourself. We were at the same starting point and left the line even though we were strolling.”
Staff member ZSJ	Supporter	Participate in discussions actively	“The experience shared is especially wonderful, and success belongs to those who are prepared.”
		Drive organizational atmosphere	“The self-organized form of full participation allows everyone to learn a lot. The implementation of the evaluation results is for both the individual and the team. The debriefing results are secondary. As long as everyone enjoys the process, personal development and growth are the most important.”
Staff member LMH	Innovation actor	Participate in innovation actively	“We need to innovate constantly to create different freshness so that customers can enjoy it. This is the value of our service.”
Supervisor CYY	Encourager	Promote positive emotions	“Staff SGH, your competition speech yesterday was excellent. You don’t need any fancy words for the competition. It’s more pleasing to say the truth.”
		Encourage subordinates to participate in job debriefing and competition	“The environment of the company allows us to rectify and learn. The development of the company requires the strength of each of us. Come on!”
Supervisor PZW	Reverse model and innovation cultivator	Dissipate and transform negative emotion	“The Competition Process is no longer a formality; I need to dissipate( myself enough to break the bureaucracy.”
		Self-reflect	“It is wrong to rely on parental control to lead the team.”
		Drive team members to reflect	“Did my failure in the competition lead to other people’s reflection?”
		Bond with team members	“ Thank you all my members for making me grow. I will get up from where I fall.”
		Nurture member innovation	“From the consensus in April, independence, claiming, to the implementation in May, the staged ground promotion, and full-staff marketing, from the perspective of customer needs, we can be seen in the surrounding areas, responsible for independent, person-time venture capital, product generation, all the efforts are worthwhile, and we look forward to bringing you a different wonderful.”
Supervisor ZJ	Innovation reflector	Repair the harmful effects of negative emotions	“Although everyone is exhausted the result is so good, everything is worth it.”
		Rethink marketing innovation	“Question 1: How to more effectively evaluate the revisit rate of tourists? Question 2: The relationship between the revisit rate of tourists and the service level of the park and the effect of marketing promotion? Question 3: What is the revisit rate of tourists in 2019?”
Deputy General Manager WXX	Inspirer	Expand the scope of individual actions	“Doing things like a leader, doing things for tomorrow, it’s the spirit of the autonomous culture.”
		Analyze problems	“Analysis of the reasons at the bottom of the system ranking: Symptoms: Insufficient sharing of failure skills; Crux: Member evaluation is not related to work; Symptoms: Information is not transferred to each member.”
		Mentor staff	“Today’s sharing is to create an open environment. Everything is brought to the table, and no complaints are below. Everyone should give real feedback.”
		Quote allusions to explain	“ In Chinese old saying……”
		Boost morale	“Good job, we have strength, we have wisdom, we have responsibilities, and let’s cheer up.”
Deputy General Manager ZZM	Encourager	Promote positive emotions	“You have made great progress, WK, you are my role model.”
		Encourage subordinates to be honest	“The positive energy of opening your heart will make you a luminary.”
		Encourage subordinates to innovate independently	“A month of planning eventually led to a successful beginning. The entire department analyzed from a strategic perspective the contact with all principals and the promotion of all members’ self-claimed claims on-site, resulting in a new record for the park’s admission rate. Their value is reflected in their efforts!”
General Manager MCY	Mentor leader	Guide and transform members’ emotions	“People’s potential is unlimited, the key is whether there is an environment to develop potential. With you in the resort, tomorrow will be better!” “The long-awaited complement post finally appeared. Congratulations to PZW’s team for their successful turnaround!
		Set up debriefing and competition rules	“I hope everyone is honest in the Debriefing Process and has a harmonious and competitive process.”
		Create an open enterprise culturally	“The environment creates people, and people are also the main body that changes the environment. I believe that everyone will cherish the environment and use continuous actions to create a greater development environment.”
		Guide members to make retrospective summaries	“I hope that PZW’s team will comment on the gains and losses of this event to help other teams reflect and learn to reverse the situation. At the same time, it is also recommended that PZW send a compliment post to praise other departments involved in this collaborative operation.”

### Narrative fragments

Narrative fragments are fundamental nodes in a narrative network that help advance the plot. Each fragment occurs between actors (employees, supervisors, and managers) and contains information about actions, focal events, emotions, etc. Over time, the language and ideas displayed in the fragment will serve as a shared, structured part of the communication pattern (Pentland and Feldman, 2008). By dismantling key fragments of events, it is helpful to understand the process of breaking through defensive reasoning at the individual level.

***From the General Individual Perspective - Stage 1.*** To illustrate this argument, we have selected narrative fragments from the case enterprise that demonstrate how individual employees overcame defensive reasoning and were motivated to engage in sincere workplace communication rather than concealing knowledge.

General Manager MCY used the Debriefing Process as the primary tool for breaking through defensive reasoning. The main purpose of the Debriefing Process is to allow employees to form the habit of being honest with each other. Under the application of reporting procedures, enterprises have formed a transformation from leadership decisions to institutional decisions.

The Debriefing Process ranks members by self-defined evaluation parameters. It allows people to self-learn and collaborate. Employees learn from one other’s debriefing speeches and share at the meeting and afterward. Through environmental stimulation and positive emotions, employees’ attention, cognition, and action expand [64], strengthening their self-learning, knowledge-sharing, and other behaviors to drive team learning and create a positive learning environment.

The reporter was selected through a lottery, which they claimed provided them the same opportunity as their superiors and enabled them to demonstrate their skills during the Debriefing Process. It gives the reporter the courage to overcome their inner negative defensive emotions and communicate freely with a positive attitude, encouraging more participants to learn from others’ strengths and fix their weaknesses, forming a self-directed learning team. According to interviews and social media comments, the Debriefing Process increased the propensity of employees to reject defensive attitudes and convey positive emotions. The following segments describe how people felt about participating in the Debriefing Process:

Participating in this consensus process is a process of learning and progress. Everyone can talk about ideas from different angles and get the final consensus. Here, synergy predominates over superficial harmony. Participation is the initial stage. (Supervisor CYY); To overcome the nervousness of coming to power is to overcome yourself (Staff member ZJX); The positive energy of opening your heart will make you a luminary (Deputy General Manager ZZM).

According to the collected data, the assessment dimensions of the company generally include primary job responsibilities, daily responsibilities, on-site interaction, discovery, and overall effects. A multidimensional system can help employees develop personally and professionally by actively seeking feedback [65]. Performance analysis can also help team members comprehend each other’s growth so they can address team issues with real-time data and targeted discussions utilizing the multidimensional indicators of the Debriefing Process.

Based on this analysis, from the general individual perspective, an open and frank organizational atmosphere has been formed in the enterprise through the change in employees’ cognition and behavior from the Debriefing Process, breaking through the defensive reasoning among members.

***From the Job Position Perspective - Stage 2.*** Open communication and knowledge sharing have boosted team cohesion by fostering positive emotions and accelerating individual performance, but the lengthy tenure of a single leader has generated new concerns regarding defensive reasoning. When the organization became autocratic and inflexible, team members became cautious and defensive in an effort to satisfy superiors.

To overcome this situation, the company has also adjusted the Competition Process. Teams at the low end of the evaluation have experienced low performance for three consecutive months. In these teams, the person in charge will be selected by the members’ vote. Deputy General Manager ZZM exampled:

Replacing the team leader breaks the closed team form, which has two main functions: first, competition for recruitment is a process of rebirth for the team; second, it opens the promotion channel for employees.

Under the dual pressures of the elimination crisis and tension between members, the introduction of the new competition rules drove the team leaders with defensive issues to finally recognize their errors, and they made a profound public reflection at the competition meeting. Supervisor PZW’s defensiveness once hampered team effectiveness. After reflecting on his behavior during the elimination crisis, he chose to alter his leadership style to be less autocratic.

The other team members led by PZW also reflected on their own mistakes and encouraged the supervisor, re-establishing team cohesion:

The failure of PZW’s competition is not a personal failure but our team’s failure. But the failure is only temporary. Our watchword is not meaningless (Staff member SNN); Our cohesion is strong, and the soul of the team has been found. I believe we will stand together through thick and thin and get better and better (Staff member SYZ).

The team’s profound reflection and passion have inspired other external teams on corporate social media platforms, and external team members have also expressed that dictatorship is not desirable and unity is the most important thing:

Managing a team in a parental way will make members passive and not work autonomously (Supervisor CYY); A single tree cannot make a forest, and unity is strength (Staff member GPN); We want to be a real team, not a gang, as long as you realize that your team will get autonomous (Staff member SFS).

Simultaneously, Supervisor WHL pointed out that the secret of leading a team is to remove self-isolation first and then disintegrate defensive reasoning.

So far, the emotions between PZW and his team members have finally changed from confrontation and negativity to understanding, comprehending, encouraging, and other positive emotions. Under the Competition Process, the emotional transmission also reversed the dilemma of defensive reasoning.

With the continuous iteration of the Competition Process, the promotion of enterprise members is determined by the personal assessment results, with top-ranked employees able to compete for management positions based on their abilities. This has enabled individuals to operate more independently, maximize their potential, and generate a large number of “grassroots heroes” within the organization. Staff such as SGH have been foundational for years. They vied for the position of team leader due to their excellent performance. In less than a month, SGH led her colleagues to a performance triumph, increasing the number of A-level employees by 9 and the average employee score by 18. SGH believes that the true objective of the competition is growth, knowledge, and ideas.

Under the action of the Competition Process, the enterprise members no longer need defensive reasoning to protect their interests. Instead, they can succeed by opening their minds and constantly breaking personal ability boundaries.

Based on the information discussed from social media platforms, managers and members have the courage to be honest and open about their mistakes, dissipate negative emotions, ruminate on the cause of the errors, and reverse negative behaviors. Individual defensive reasoning has been conquered.

***Promote Innovation and Cultivation After Defense Breakthrough.*** Supervisor PZW overcame his defensiveness and consciously instructed team members and newbies in our follow-up. He expanded target client groups, and members autonomously used multi-way promotion tactics to develop services, products, and marketing. Methods are in Table 4. Park B helped them open and operate that year. Park A had 327 visitors, whereas Park B had 2,000 and an average stay of 5 h. 800 passengers poured instantly, peaking at 1,600.

This accomplishment has been validated by numerous parties, and team members relished the process of working independently, viewing innovation as their tenacious pursuit; for example: Always innovate, create a different sense of freshness, and let customers enjoy it, this is the value of our service (Staff member LMH), and Each part uses the weekend break to promote in the surrounding area in the form of independent points to attract a large number of tourists. Our hard work has paid off due to our concerted efforts (Staff member SXY).

External team members also believe that PZW’s team members are positive and enthusiastic about pursuing innovation independently: From the post, it can be seen that the park has an autonomous team, and everyone has a clearer and more positive spirit of autonomy (Supervisor CYN).

Even though this marketing achieved gratifying results, the members did not forget to summarize, reflect, and remind themselves not to be proud of good results but to pay attention to the next move.

This analysis of the successful case of PZW’s team reversal shows that there are two main aspects to their work: breaking through the defensive reasoning at the individual level and cultivating the innovative spirit of the team.

**Table 4:** Supervisor PZW and his team members’ work reversal practices

Work Aspects	Procedure	Specific actions
Breaking through defensive reasoning at the individual level	Personal reflection	Openly engage in comprehensive personal reflection
	Team reflection	Reflect on your behavior
	Reshaping team cohesion	Make a resurrection guarantee contract for the person in charge, and deeply bind individual performance with team performance
Cultivating innovation	Rulemaking	Enact modular division of labor, based on system evaluation and self-claimed by members
	Motivational approach	E***nergize*** with points as value accumulation
	Self-learning	Use the non-working time to learn other skills
	Knowledge sharing	Share knowledge within the team; rapidly integrate new employee learning skills
	Collaboration with external teams	Support work (external team members)
	Inspiring out-of-role behavior	Use the non-working time to part-time other positions to promote the implementation of the event
	Market developing	Business dock with target customer groups to form long-term cooperation
	Forward-thinking	Check safety facilities in advance
	Reflection after action	Summarize experience and ask questions

### Narrative networks of breaking through the defensive reasoning process

To construct a more detailed narrative, we have compiled a complete list of focal events overcoming defensive reasoning in the enterprise over the case study period, shown in Table 5.

**Fig. 1:**
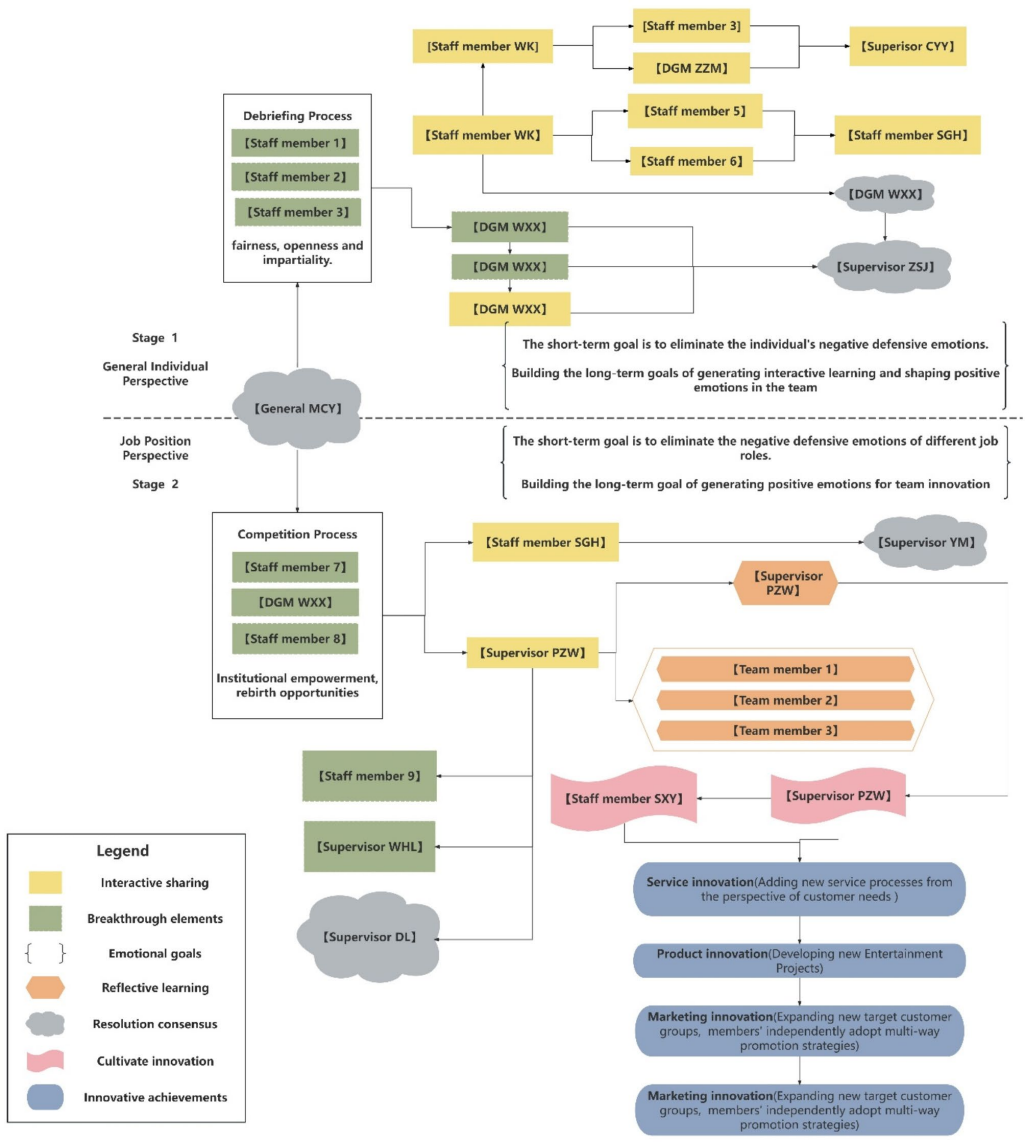
A narrative network analysis diagram of breaking through defensive reasoning at the individual level drive to innovation (simplified version)

The simplified narrative network analysis diagram of reasoning is shown in Fig. 1 of the main text. This diagram highlights key elements of the breakthrough process using different colors and shapes, including interactive sharing, breakthrough elements, emotional goals, reflective learning, resolution consensus, innovation cultivation, and innovative achievements. In the simplified version, only the employee hierarchy and participation codes are displayed, while the full diagram and detailed dialogue information can be found in the appendix.

Stage 1 in the diagram illustrates the process of breaking individual defense mechanisms from a general individual perspective. The company primarily utilizes the Debriefing Process to help employees understand the organization’s open system, focusing on conveying key values such as fairness, openness, and impartiality. By offering equal opportunities for participation, employees are encouraged to speak up and empowered to solve problems.

Stage 2 shows the process of overcoming defense mechanisms from a job position perspective. This stage addresses defensive behaviors, such as sluggishness and superficial submission, triggered by supervisors’ controlling tendencies and closed-off attitudes. The solution employed in the case company is the Competition Process, which helps break down hierarchical communication and promotion barriers between supervisors and subordinates. The central message conveyed here is the empowerment signal and rebirth opportunities embedded within the institutional environment. By providing equal institutional authority, employees are freed from the need to seek approval from superiors, gaining greater control over their own work. Additionally, positive emotions and information are spread through the social media platform. The diagram emphasizes the process of information exchange, emotional transmission, open-mindedness, and the completion of autonomous innovation among participants.

**Table 5:** Descriptions of breakthrough defensive reasoning event categories

Feature	Focal event of breakthrough defensive reasoning			Effect
	Event node	Date	Detailed description	
Stage 1: Refusal to be honest	Monthly Debriefing Process	April 25, 2018	Debriefing according to results is changed to a random lottery, showing equal opportunities for everyone Staff enthusiasm is mobilized via team debriefing Persistent problem-seeking behavior is subverted; all staff are guided to rethink and find motivation for results	Defensive reasoning is conquered at the general individual level
	Changes in employees	April 27, 2018	Members are proactive and team cohesion increases New role model	
Stage 2: Leader behavior: a strong desire for control and reluctant to listen to multiple voices Staff behavior: sluggish and superficially submissive	Monthly Competition Process unsuccessful	February 17, 2019	New mechanism of three reversal chances for death–rebirth Team leader introspection triggers the collective reflection of team members	Defensive reasoning is broken through at the job position level
	Status return visit	March 5, 2019	The circle structure is broken Concept of cultivating people, developing new talents in collaboration	
	Cultivation of innovation	June 2, 2019	Supervisors use the rules and enterprise social media platform to foster the development of members. Intentionally, the status of team members is reversed, innovation is achieved independently, and the new marketing strategy establishes a record	Members are motivated to innovate

## Discussion

Breaking through defensive reasoning at the individual level and transforming it into a foundation for innovation requires a multifaceted approach. Our analysis reveals how structured processes, institutional environments, and digital platforms collaboratively address defensive reasoning while fostering creativity and team synergy.

At the individual level, structured debriefing processes alleviate defensive emotions like fear of criticism by fostering open communication and fair performance evaluation. This builds trust, encourages shared learning, and shifts employees from passive to proactive engagement, laying the groundwork for collaboration and creativity. Positional challenges are addressed through competitive processes that emphasize fairness and transparency. By reframing competition as a growth opportunity, employees are motivated to learn and cooperate rather than resist. Leaders undergoing re-competition serve as examples of rebuilding trust and fostering team cohesion, driving individual and group-level innovation.

The institutional environment enhances these efforts by emphasizing openness, autonomy, and adaptability. Clear guidelines and reward systems reduce fear and insecurity, encouraging employees to take risks and view setbacks as learning opportunities. This structural support transforms defensive behaviors into proactive contributions, reinforcing a culture of creativity. Internal social media platforms amplify these initiatives by enabling transparent communication, collaboration, and feedback. Virtual role transformations further enhance innovation by allowing employees to experiment with diverse perspectives, fostering adaptability and creative problem-solving.

Together, these strategies align individual and organizational goals, transforming defensive reasoning into a catalyst for innovation. By addressing emotional, cognitive, and structural barriers, organizations can build a resilient, collaborative, and creative culture that drives sustained growth and improvement.

### From defense breakthrough to innovation cultivation

**Fig. 2:**
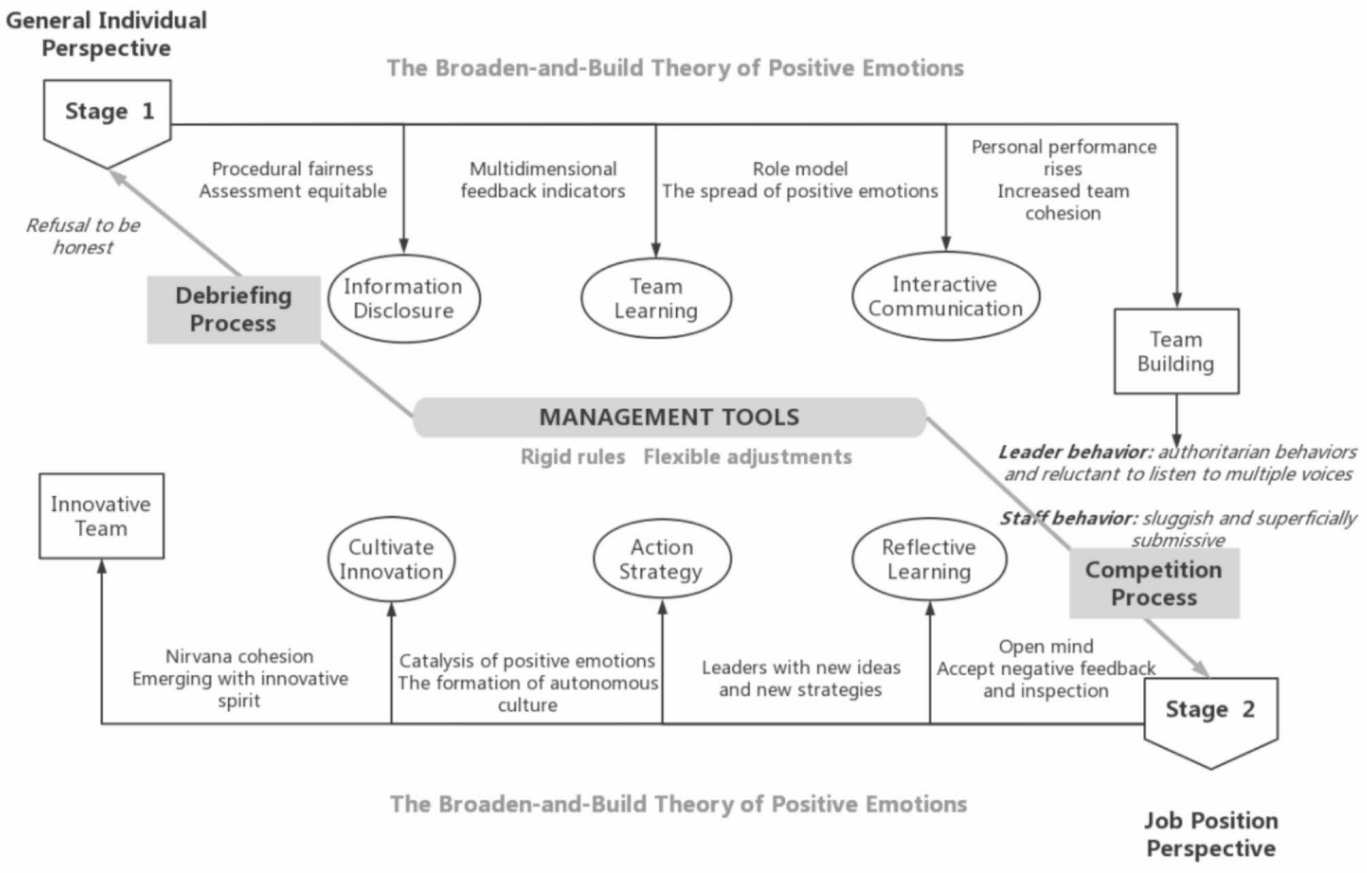
Path process of breaking through defensive reasoning to cultivate innovation

As mentioned, although some studies have discussed the breakthrough method of organizational defense, the breakthrough process and elements of defensive reasoning at the individual level have not been explained to a great extent. By analyzing focal events from a general individual perspective and a job position perspective in our research case, we have shown the process by which companies use management tools to break through individual-level defensive reasoning and then cultivate innovation, as shown in Fig. 2.

***General Individual Perspective—Debriefing Process.*** The setting of the Debriefing Process is used to break through the defensive reasoning that is characterized by refusal to be honest, and the short-term goal is to eliminate the individual’s negative defensive emotions, building the long-term goals of generating interactive learning and shaping positive emotions in the team.

The enterprise’s social media network displays Debriefing Process evaluation metrics and performance information. Fair methods and outcomes satisfy the psychological needs of employees, enhance their personal beliefs and sense of control, and reduce their reticence [66]. Complete trust will encourage employees to consider one another’s interests throughout all work interactions [67]. The reward system fosters positive feelings among employees, thereby transforming their behavior from passively open to actively forthright.

In a low-transparency workplace, individuals with less information will perceive greater uncertainty and will be more likely to be hostile, recalcitrant, and defensive. This organization provides straightforward information channels, an open work environment, and autonomous promotion channels. The Debriefing Process’s multidimensional evaluation strategy reduces job insecurity by providing explicit and specific job feedback, allowing employees to quickly receive feedback and gain knowledge. Moreover, promoted workers can function as role models. Members dismantle defensive reasoning through the sharing and improvement of information. This accelerates the exchange of knowledge and information, the improvement of performance, team cohesion, and the dissemination of positive emotions. Thus, the Debriefing Process enables employees to evaluate the company’s impartiality in public and to share and learn.

***Job Position Perspective—Competition Process.*** The Competition Process is used to break through the defensive reasoning generated by the different job positions of enterprise members. The short-term goal is to eliminate the negative defensive emotions of the different job positions, and building the long-term goal is to create positive emotions for team innovation.

Enterprise competition is beneficial, educational, and non-destructive. For enterprise members, the elimination crises and interpersonal stress caused by competition have changed. Instead, enterprise employees are receptive to learning and advancement, regardless of the failure of the competition. Knowledge-sharing promotes positive emotions and innovation [68], and the case company’s rotation system fosters self-learning, knowledge-sharing, and emotional connections. For those in charge who need to reapply for their previous position due to unsatisfactory debriefing results, such as Supervisor PZW, success in the competition depends not only on the extent of their reversal but also on the commitment of team members to work together (members sign the reversal commitment contract and attend the contract ceremony for the person in charge). The Competition Process is intended to reinvigorate a competitor’s fighting spirit, enhance cooperation, and improve mood. The emotional behavior of supervisors influences employee development and positive affect, which stimulates creativity [69]. Thus, the supervisor’s change in management style will result in greater work autonomy, learning and sharing, and audacious innovation, replacing the team’s negative defensive emotions with positive emotions and innovation spirit.

General Manager MCY mentioned that:

During the Competition Process, with the rules and information transparency, everyone should compete openly, so people don’t dare to show their inner evil, and they all speak with justice.

It entails using fairness and benevolence to maximize the influence of the populace and the rules. The way to solve problems in the Competition Process is to rely on everyone’s consensus, and the key to re-competition is by tapping into the positive energy within employees to make decisions, which is more beneficial than detrimental to the development of the enterprise.

Finally, the two management techniques collaborate to alter employees’ emotional states and action plans and teach them to communicate openly and truthfully to overcome their defensive reasoning. Such an organizational culture consistently stimulates and encourages employees’ positive emotions, cultivates their innovative spirit efficiently and consistently, and fosters an organized and collaborative creativity environment in the enterprise.

From the general individual perspective, the Debriefing Process reduces defensive reasoning by addressing negative emotions and promoting trust, transparency, and constructive feedback, which enhances learning and team cohesion. From the job position perspective, the Competition Process breaks down defensive reasoning linked to job roles, fostering healthy competition, collaboration, and positive emotions that inspire innovation and strengthen team dynamics.

### Institutional environment—guidance and transformation of emotions

The case enterprise’s environmental system model exhibits five essential characteristics that effectively transform individual defensive reasoning into positive emotions and foster innovation. First, a networked hierarchical structure promotes cross-functional collaboration and flattens traditional boundaries, enhancing adaptability and responsiveness. Second, open and transparent information ensures all employees have equal access to critical data, reducing uncertainty and fostering trust. Third, transparent procedural fairness and rules create an equitable environment where employees feel secure and motivated to contribute. Fourth, positive error management encourages employees to view mistakes as learning opportunities, reducing fear of failure and stimulating creativity. Finally, a dissipative organizational culture actively breaks down rigid behaviors and resistance to change, fostering adaptability, creativity, and a shared commitment to innovation. Together, these elements create a supportive environment where defensive reasoning is replaced by a culture of trust, openness, and collaborative growth.

Institutional factors (or organizational context) can provide meaning and understanding in situations and shape members’ beliefs and behaviors by providing normative templates to validate specific behaviors and sanction individual behaviors [70]. People continuously scan their environment to detect and assess changes, and emotional responses occur when individuals perceive experiences related to their goals to correlate with or affect their self-concept and normative systems [71]. Institutional environments influence emotions by shaping, regulating, and managing emotional expressions and responses. Within a given societal institutional framework, emotional expression and processing are subject to explicit norms and constraints. These norms may be established through social conventions, values, and cultural cognitions, impacting how individuals perceive, express, and cope with emotions((Ashforth, 1995 #703). For instance, certain institutional contexts may encourage the expression of specific emotions, while others may impose limitations. Institutional environments also guide and influence emotions through organizational structures, policies, regulations, and social customs. Thus, the role of institutional environments in emotions can affect individuals’ emotional experiences, expressions, and management strategies [72]. The enterprise’s system opposes the bureaucratic focus on openness, and employees no longer had negative emotions of worry or embarrassment due to fear of mistakes at work but instead gained more autonomy and rewards, inspiring them to generate more positive emotions. Emotions explain how and why individuals respond to issues of fairness and provide insight into their behavior [73]. According to our case analysis, the enterprise employees broke out of the dilemma of being promoted according to the opinions of their superiors. Instead, their career path depends on their ability. Employees trust the enterprise’s assessment system and receive positive emotional feedback to satisfy their sense of security and trust. Simultaneously, because the system is mandatory and punitive, employees will adopt compliant strategies even if they recognize the system’s ideas or rules. This will gradually transform employees’ passive defensive reasoning into active ones, influencing their behavior. According to the broaden-and-build theory, positive emotions broaden individuals’ cognitive scope and stimulate their imagination and innovation ability [36], which means employees are more willing to perform actively at work and break through personal cognition and action boundaries. Individuals’ desire for reward, even if they feel increasing pressure (a negative emotion), will still produce positive results [74]; that is, the motivational effect of the competition mechanism on employees.

Emotions regulate cognition and behavior, provide vitality and direction, and aid in understanding corporate innovation management [75]. Positive emotions assist members in overcoming negative emotions and finding a constructive solution when they use defensive reasoning to avoid humiliation or errors. The effectiveness of organizational work objectives will be influenced by fluid organizational structures [76], resource availability [77], organizational climate or culture [78], and various supportive aspects of innovation [79]. Thus, the enterprise’s environmental system model satisfies the development requirements of all employees, shapes their collective efficacy beliefs at work, assists them in overcoming defensive reasoning, and fosters a positive perspective on innovation accessibility and confidence.

The institutional environment of the case company, with its emphasis on transparent information flow, fair procedures, and autonomy, actively reduces defensive reasoning by promoting positive emotions and enhancing employees’ sense of safety and trust. By providing clear standards and rewards, and aligning with the broaden-and-build theory of positive emotions, the organization fosters cognitive flexibility and creativity, enabling employees to overcome defensive barriers and engage more proactively in innovative behaviors.

### Social-media platform—diffusion and spread of positive emotions

Some of the data cited in this study are from enterprise social media platform sources. The platform has both routine social and management-program functions, including daily information release and communication and displaying staff and team performance data. Employees can discuss and make decisions related to management on this platform. Employees use their real names to discuss work projects, organizational goals, policies and procedures, work-related information and documents, and hot management events on the platform. The company’s social media platform is accessible, impartial, and equitable, and it enables members to connect.

In virtual environments, individuals have the opportunity to convey information about community characteristics, such as rules and values, through various means, including text, images, and links [80]. Compared to traditional modes of information dissemination, virtual environments eliminate the trade-off between information richness and dissemination reach. This facilitates potential members to better understand specific virtual groups, thereby reducing information asymmetry. The Internet enables virtual communication among members to be more uninhibited, creative, and direct compared to face-to-face interaction [81]. People’s emotional response comes from the perception of the environment and evaluation [71], the fair environment and just decision-making provided by the enterprise are the soil for cultivating positive emotions in the organization. Because the enterprise’s social-media platform’s rules for publishing and discussing information are constrained by the system with a high degree of openness and transparency, allowing employees to fully feel procedural, distributive, interpersonal, and informational justice, it diffuses members’ positive emotions. Previous studies found that expressing positive emotions affirms a pleasant relationship [82], strengthens social ties [83], reinforces group attachment [84], increases trust [85], and improves the group’s emotional atmosphere [86]. Therefore, the company’s social media platform has two important functions: First, through the diffusion of positive emotions, to increase the possibility of the members who are afraid to express themselves but are eager to take the first step. Second, through the spread of positive emotions, to convey the message that progresses, only when we open our minds can we overcome our defenses.

There are also individual-level defensive phenomena on social media platforms, such as the justification of concealed information and concealing techniques, but institutional rules prohibit them. Employees can readily access one another’s employment information and discuss workplace management issues on social media platforms. Information transparency provides employees with numerous hints for reducing uncertainty and ambiguity and promptly understanding what other counterparts know, thereby encouraging them to seek feedback [87] and share their expertise. The beneficial and reciprocal trust connection between members lowers the cost of information communication and reduces the unpleasant feelings induced by defensive reasoning connected to avoidance and withdrawal [73].

Concurrently, the task-oriented function of the enterprise’s social media platform improves employees’ positive moods by rewarding them with points to stimulate their innovativeness. Thus, the case enterprise’s social-media platform helps employees to open their minds, overcome their defenses, and stimulate their spirit of innovation through the diffusion and spread of positive emotions. Social media platforms rely on institutions to ensure the justice and impartiality of the organization, as well as on the expansion of positive emotions to build members’ resources.

In sum, the case company’s social media platform integrates both social and managerial functions, promoting transparency, fairness, and open communication among employees, thus fostering a fair and emotionally positive environment. This platform encourages knowledge-sharing, reduces defensive reasoning, and enhances innovation by enabling task-oriented interactions and rewarding employees, ultimately promoting collaboration and the spread of positive emotions through the broaden-and-build theory of positive emotions.

### Virtual-reality multi-role transformation—Stimulation and promotion of positive emotions

Through their communications on social media platforms, members assume unique virtual-identity roles, which play a significant role in the positive emotions of enterprise members. Table 6 displays the categories of roles extracted from the case data and their effects.

Table 6 outlines the transformative impact of virtual role reversal on workplace dynamics, emphasizing its ability to break down defensive reasoning and foster positive emotions that drive organizational innovation. The roles and their effects demonstrate how virtual identities create a supportive environment for emotional and behavioral change. Below is a detailed analysis of each role category and its broader implications:

Staff-level roles, such as Bench-marker, Exemplary, Supporter, and Innovation Actor, focus on inspiring positive emotions, fostering collaboration, and showcasing innovative practices, creating a culture of achievement and shared learning. Managerial roles, including Encourager, Reverse Model, and Innovation Incubator, work to dismantle defensive reasoning, reshape team dynamics, and cultivate innovation by guiding teams toward shared goals. Leadership roles, like Inspiration Guider and Mentor Leader, drive organizational progress by fostering openness, emotional growth, and team-wide innovation through supportive and visionary leadership.

The virtual role framework systematically breaks down defensive reasoning, replacing it with trust and collaboration. By promoting positive emotions, it enhances cognitive and social resources, aligning individual growth with organizational goals. Leaders act as emotional strategists, guiding employees to transform challenges into opportunities, fostering a resilient culture of continuous learning and sustained innovation.

Lin, Qu [88] noted that group emotions and behaviors are influenced by each other, with the strength of the influence increasing with the similarity of the actors. The company’s social media platform incorporates workplace and virtual responsibilities, enabling cross-departmental and cross-level collaboration. Members of virtual communities develop a mindset of “must-do” and “willing-to-do” under normative pressure [89]. In the context of social facilitation, members of virtual communities are influenced by the expectations of their role partners (e.g., fellow members), which also affects the willingness of external members to participate compared to formal relationships. In virtual communities, higher levels of group norms lead members to be more inclined to engage in contributory behaviors, thereby eliciting positive emotional experiences such as excitement, optimism, or anticipation [81].

According to the broaden-and-build theory [64], positive emotions provide cognitive resources and adaptability to enhance spontaneous thoughts, impulses to act, and perceptions. Positive emotions assist participants and observers in the development of psychological resources applicable to a variety of roles. Positive emotions can help individuals grow and develop their abilities for self-transcendence [90]. In other words, the positive emotions induced by employee job changes may assist individuals in recognizing defensive reasoning, overcoming it, and innovating.

That is, the virtual-reality multi-role transformation on social media platforms helps dismantle defensive reasoning and foster positive emotions by allowing members to assume diverse roles that promote collaboration and innovation. These roles, ranging from staff-level to leadership, create a supportive environment that enhances emotional and cognitive resources, aligning individual growth with organizational objectives, and driving continuous learning and innovation through the broaden-and-build theory of positive emotions.

**Table 6:** *The effect of role reversal*

Workplace role	Virtual role	Purpose	Emotional effect	Effect
Staff	Bench-marker	Establish a typical positive stimulus	Stimulate positive emotions	Become a role model in the company
Staff	Exemplary	Inspire and form a social promotion role	Stimulate and promote positive emotions	Involve more people
Staff	Supporter	Inspire and form a social promotion role	Promote positive emotions	Involve more people
Staff	Innovation actor	Take the lead in innovation	Expand positive emotions	Demonstrate enterprise innovation practice for the first time
Employee, deputy general manager	Encourager	Lift individual isolation and encourage innovation	Channel negativity Promote positive emotions	Eliminate individual defensive reasoning and create a positive organizational climate
Director	Reverse model	Eliminate individual defensive reasoning	Channel negativity	Promote personal growth and reshape team cohesion
Director	Innovation incubator	Stimulate the innovative enthusiasm of employees	Expand positive emotions	Cultivate innovative teams to achieve innovative performance
Deputy general manager	Inspiration guider	Eliminate individual defensive reasoning and encourage employee innovation	Channel negative emotions Promote positive emotions	Improve team performance and gain innovative performance
General manager	Mentor leader	Form an open and dissipative self-organizing environment	Construct positive emotions	Promote staff growth, and increase team progress

## Conclusion

This study illustrates how organizational factors such as institutional environment, social-media platforms, and virtual-reality role transformations help overcome defensive reasoning and foster innovation. It shows how negative emotions and defensive behaviors can be transformed into positive, creativity-enhancing actions through emotional and cognitive shifts. By tracking longitudinal cases, we categorize the characteristics and breakthrough processes of individual defenses into the general individual perspective and the job position perspective, thereby enriching the perspectives and conclusions of prior research.

An enabling institutional environment plays a vital role in guiding emotional transformation. It fosters openness and adaptability, helping employees replace fear and resistance with trust and optimism. Social-media platforms facilitate the spread of positive emotions by promoting transparency and open communication. By sharing achievements and providing feedback, these platforms create a culture of fairness and mutual respect, reducing defensive behaviors and encouraging collaboration. Virtual-reality environments allow employees to explore different roles and perspectives, fostering empathy and collaboration. These settings stimulate positive emotions like excitement and curiosity, which carry over into real-world behaviors, enhancing creativity and reducing defensiveness. Digital management tools support transparency and fairness in organizational communication. Together with other organizational elements, they create a culture of openness that drives creativity and progress. In conclusion, overcoming defensive reasoning requires a combination of emotional guidance, role transformation, and transparent digital tools. These elements collectively create an environment where employees can move beyond defensiveness, collaborate openly, and contribute to a culture of continuous innovation.

This study offers significant theoretical contributions by providing a comprehensive examination of individual defensive reasoning through narrative network analysis. Firstly, we present a detailed narrative display of the roles, actions, narrative fragments, and key interactions involved in overcoming defensive reasoning, supplemented by graphical representations. This comprehensive theoretical model expands on previous research by distinguishing defensive reasoning into the general individual perspective and the job position perspective, highlighting the evolution of individual defenses and corresponding strategies in case development. In addition, this paper conducts an in-depth analysis of the various forms of individual defenses from a narrative network analysis approach, drawing on real-world management scenarios. Through a detailed case study of a single longitudinal example, the paper examines the management practices that breakthrough organizational defenses. This process builds a complete narrative network of overcoming individual-level defenses, offering a more comprehensive and systematic theoretical framework for research on individual defenses from different perspectives. This provides valuable insights and reference points for the study of defensive reasoning in management contexts.

Secondly, this research employs the broaden-and-build theory of positive emotions to elucidate their crucial role in overcoming defensive reasoning within organizations. It demonstrates how digital management tools and virtual communities indirectly yet effectively transform negative emotions into positive ones, benefiting organizational development. Defensive reasoning, which often originates from negative emotions, can be mitigated by understanding and addressing individual psychological needs. By strategically using digital management tools to guide and regulate emotions, negative feelings can be reframed into positive ones, thus enhancing both individual and organizational efficiency while fostering innovation. Moreover, this study not only validates previous theoretical conclusions on organizational defense but also introduces new theoretical insights. It suggests that negative emotions, typically seen as barriers to progress, can, become powerful tools for breaking through defenses when approached correctly. The key lies in how these emotions are handled—by fostering an open-minded approach and using them constructively, organizations can turn potential obstacles into opportunities for growth and development.

Thirdly, in the context of digital management, this study addresses the phenomenon of virtual-real role transitions among employees in virtual communities formed through corporate social media platforms. When employees discuss tasks and events on these platforms, their identities often blur, freeing them from the constraints and pressures of their real-life roles. These roles fluidity enables individuals to express their views more openly, release negative emotions such as fear and dissatisfaction, and ultimately foster positive emotions through knowledge sharing and emotional connections within the virtual community. This process effectively reduces individual defensive reasoning and promotes innovation, demonstrating the transformative potential of digital management tools in modern organizational environments. Furthermore, the shift between virtual and real identities in the digital age can alleviate the fear of speaking the truth often caused by defensive reasoning, enriching the individual’s multi-faceted identity roles. It accelerates the work boundaries and enhances employees’ freedom under the empowerment of the organization’s digital framework. However, it is also crucial to recognize that this identity transition may introduce new role pressures and conflicts. Organizations must be prepared to address these challenges, ensuring that the benefits of virtual role fluidity do not result in new sources of tension or misalignment within the workplace.

In conclusion, this study holds significant practical implications for corporate management. Firstly, it is crucial for managers to promptly identify and address negative emotions such as resistance, fear, and dissatisfaction among employees to mitigate the emergence of defensive reasoning. Managers should not avoid or exacerbate defensive behaviors but should instead recognize them, understand the employees’ genuine developmental needs, and take proactive measures to address and improve these behaviors. This approach prevents the transformation of individual defensive reasoning into defensive routines. To prevent defensive reasoning, leaders need to maintain an open mind, demonstrate honesty, and establish transparent, fair, and inclusive workplace norms.

Secondly, companies should consider individual emotions. Managers should learn to strategically utilize digital management tools to guide employees in transforming negative emotions into positive ones. By fostering positive emotions among team members, managers can enhance interpersonal trust, self-transcendence, and openness, thereby developing psychological resources that ultimately stimulate inventiveness within the organization.

Thirdly, managers need to recognize the hidden potential of digital management tools and virtual communities. By leveraging big data, cloud analytics, and artificial intelligence, managers can analyze and predict employees’ psychological needs, providing targeted guidance. Virtual communities formed through these digital tools enable employees to share emotions, identity recognition, and organizational knowledge, thereby facilitating the achievement of organizational goals.

This study also has some limitations. First, defensive reasoning, a product of people’s automatic subconsciousness, is impacted by the external environment and other people’s conduct owing to the diversity of people’s experiences and viewpoints. Leaders should objectively examine the circumstances to choose the most effective management methods because behavioral responses are random. Second, since this study has a single-case enterprise as its analysis object, the process path for overcoming individual defensive reasoning may not be universal and should be tested with a large sample.

In terms of implications, the approach to breaking individual defense mechanisms demonstrated in this study not only benefits the tourism industry but also offers valuable insights for other sectors. For instance, in healthcare, where medical staff often face high pressure and emotional burdens, defensive reasoning may provide temporary relief from stress, but it can also obstruct effective communication, ultimately affecting the quality of care. By adopting digital tools and virtual role transformations similar to those discussed in this study, healthcare institutions can cultivate an open communication environment, dismantling emotional defenses inherent in traditional hierarchical structures. This would encourage medical professionals to share experiences, discuss complex cases, and, as a result, enhance teamwork and innovation. Additionally, transparent performance feedback and open information channels can improve psychological safety among staff, reducing negative emotions associated with mistakes or decision-making errors.

In the manufacturing sector, implementing social platforms and role-switching mechanisms could increase employee engagement and autonomy, improve interdepartmental communication, break down silos, and facilitate timely information exchange, all of which contribute to enhanced innovation. In the technology industry, where innovation pressure is particularly high, employees may develop defensive reasoning due to fear of failure, which can stifle creativity. The creation of open, transparent communication platforms, coupled with virtual role transformations, can help employees overcome this fear, strengthen team collaboration, and foster an innovation-driven culture. In such environments, the dissemination of positive emotions encourages employees to share ideas and technical knowledge, thereby accelerating technological breakthroughs and product innovation.

However, it is crucial to acknowledge the potential risks associated with the use of digital tools, including privacy concerns, technological barriers, and over-reliance on tools that may neglect individual needs. While industries strive to enhance productivity and foster open environments through digital tools, it is essential to maintain a balance between the application of technology and the consideration of employees’ emotional and psychological experiences. In conclusion, by leveraging digital tools to break traditional hierarchical and role-based constraints, organizations can cultivate a more open, trusting atmosphere that alleviates individual defense mechanisms, thereby stimulating innovation and positive emotions. The successful application of these methods is contingent upon the specific characteristics of each industry, organizational culture, and the unique needs of employees, suggesting that their application should be tailored and optimized for each context.

Existing research on defense mechanisms at the organizational level has primarily focused on their negative impacts on organizational learning and effective organizational change, as well as the influence of defense actions at the employee level. For instance, defense behaviors triggered by employee responses to customer complaints have been explored, specifically looking at how a customer-oriented management atmosphere influences employee reactions to complaints and subsequent work performance. While these studies have addressed the negative consequences of defense mechanisms, the classification of such mechanisms remains underdeveloped, and the related solutions lack a systematic and targeted approach. Given the limitations inherent in the single-case data sample, future research could expand upon this study by incorporating cross-industry and cross-geographical comparisons to enhance the generalizability of its findings. Specifically, it would be valuable to investigate how the interplay between defensive reasoning and innovation varies across different sectors, such as healthcare and manufacturing. This would help assess whether the observed dynamics are sector-specific or more universally applicable.

Additionally, future studies should consider incorporating paired data on individual defensive reasoning and leadership decision-making. Collecting both individual-level defense data and corresponding leadership decision data would facilitate a more nuanced understanding of the decision-action relationship, thereby clarifying how managerial interventions influence employee behavior. This approach would contribute to a deeper understanding of how defensive reasoning evolves and how leadership strategies can effectively mitigate its impact.

An important direction for future research involves examining organizational-level defense mechanisms, particularly in the context of digital transformation. Researchers could explore how defense practices evolve as organizations undergo digital transitions, identifying the impact of digital tools and role transformations on organizational defense behaviors. Future research could focus on examining whether various digital transformation tools have a masking effect on defensive reasoning, and conduct comparative studies to explore how these tools differ from traditional organizational defense mechanisms in established industries. Such comparisons would provide valuable insights into how digital transformation shapes the emergence and nature of organizational defense practices.

Finally, expanding the theoretical lens would offer new insights into the underlying mechanisms of defensive reasoning. Integrating perspectives such as Prospect Theory and Event System Theory could provide a deeper understanding of how defensive behaviors are triggered and evolve within uncertain and dynamic environments. These frameworks could help explain the psychological and situational factors contributing to defensive reasoning, thus enriching strategies to address and overcome these barriers. Longitudinal studies across multiple organizations would also enable researchers to validate and extend the findings of this single-case study, offering a more robust theoretical framework for understanding the long-term dynamics between defensive reasoning and innovation.

## Electronic supplementary material

Below is the link to the electronic supplementary material.

Supplementary Material 1

## Data Availability

No datasets were generated or analysed during the current study.

## Footnotes

1 Debriefing Process. In this process, representative employees of each module are required to share, without reservation, their successful and failed experiences with the audience, according to the multidimensional assessment system. Based on General Manager MCY’s concept, even if the performance score is low, the debriefing score will still be high if the sharing is thorough. However, if the team leader’s performance is unsatisfactory three times consecutively or four times in six months (three times consecutively), they will face possible salary reduction or even contract termination, only through re-competition can there be a chance of resurrection. Competition Process. The promotion of an employee is determined by the individual’s performance and multidimensional assessment results, not by the leadership. This is allowed even if employees want to apply for other department positions. In addition, those supervisors who fail in the competition process and need to participate in the competition again are required to have team members’ guarantees and joint contracts to re-compete.
