# Supplementary material for: From defensive reasoning to innovation: how digital tools foster positive emotions in organizations
Source: BMC Psychol. 2025 Feb 20;13:146. doi: 10.1186/s40359-025-02486-6 (PMC11843981; doi:10.1186/s40359-025-02486-6)
Supplement: Supplementary file 1 — Supplementary Material 1 [file 40359_2025_2486_MOESM1_ESM.docx]

SUPPLEMENTARY FILE

**FIGURE 1** A Narrative Network Analysis Diagram of Breaking Through Defensive Reasoning at the Individual Level Drive to Innovation

**
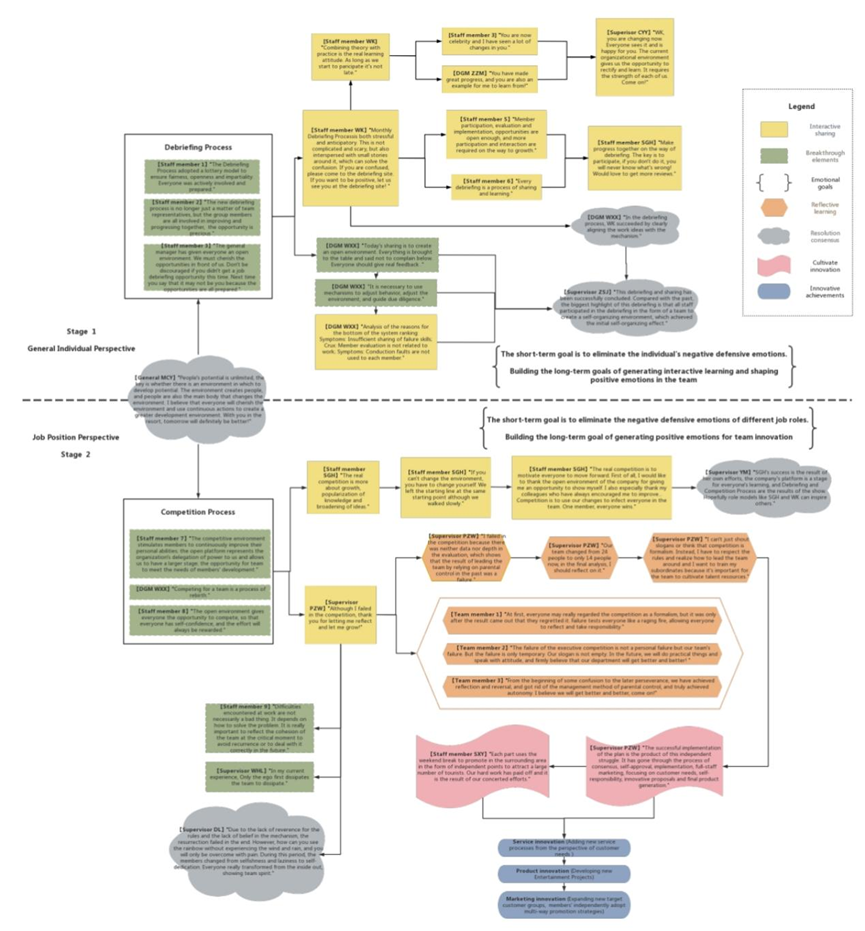
**

This diagram highlights key elements of the breakthrough process using different colors and shapes, including interactive sharing, breakthrough elements, emotional goals, reflective learning, resolution consensus, innovation cultivation, and innovative achievements.

Stage 1 in the diagram illustrates the process of breaking individual defense mechanisms from a general individual perspective. The company primarily utilizes the Debriefing Process to help employees understand the organization’s open system, focusing on conveying key values such as fairness, openness, and impartiality. By offering equal opportunities for participation, employees are encouraged to speak up and empowered to solve problems. The detailed quotation is as follows:

Debriefing Process：

【Staff member 1】"The Debriefing Process adopted a lottery model to ensure fairness, openness and impartiality. Everyone was actively involved and prepared."

【Staff member 2】"The new debriefing process is no longer just a matter of team representatives, but the group members are all involved in improving and progressing together, the opportunity is precious."

【Staff member 3】"The general manager has given everyone an open environment. We must cherish the opportunities in front of us. Don't be discouraged if you didn't get a job debriefing opportunity this time. Next time you say that it may not be you because the opportunities are all prepared."

[Staff member WK] "Combining theory with practice is the real learning attitude. As long as we start to participate it's not late."

[Staff member 3] "You are now celebrity and I have seen a lot of changes in you."

【DGM ZZM】"You have made great progress, and you are also an example for me to learn from!"

【Supervisor CYY】"WK, you are changing now. Everyone sees it and is happy for you. The current organizational environment gives us the opportunity to rectify and learn. It requires the strength of each of us. Come on!"

【Staff member WK】"Monthly Debriefing Processis both stressful and anticipatory. This is not complicated and scary, but also interspersed with small stories around it, which can solve the confusion. If you are confused, please come to the debriefing site. If you want to be positive, let us see you at the debriefing site! "

【Staff member 5】"Member participation, evaluation and implementation, opportunities are open enough, and more participation and interaction are required on the way to growth."

【Staff member 6】"Every debriefing is a process of sharing and learning."

【Staff member SGH】"Make progress together on the way of debriefing. The key is to participate, if you don't do it, you will never know what's wrong! Would love to get more reviews."

【DGM WXX】"Today's sharing is to create an open environment. Everything is brought to the table and said not to complain below. Everyone should give real feedback ."

【DGM WXX】"It is necessary to use mechanisms to adjust behavior, adjust the environment, and guide due diligence."

【DGM WXX】"Analysis of the reasons for the bottom of the system ranking: Symptoms: Insufficient sharing of failure skills; Crux: Member evaluation is not related to work; Symptoms: Conduction faults are not used to each member."

【DGM WXX】"In the debriefing process, WK succeeded by clearly aligning the work ideas with the mechanism."

【Supervisor ZSJ】"This debriefing and sharing has been successfully concluded. Compared with the past, the biggest highlight of this debriefing is that all staff participated in the debriefing in the form of a team to create a self-organizing environment, which achieved the initial self-organizing effect."

Stage 2 shows the process of overcoming defense mechanisms from a job position perspective. This stage addresses defensive behaviors, such as sluggishness and superficial submission, triggered by supervisors' controlling tendencies and closed-off attitudes. The solution employed in the case company is the Competition Process, which helps break down hierarchical communication and promotion barriers between supervisors and subordinates. The central message conveyed here is the empowerment signal and rebirth opportunities embedded within the institutional environment. The detailed quotation is as follows:

Competition Process:

【Staff member 7】"The competitive environment stimulates members to continuously improve their personal abilities; the open platform represents the organization's delegation of power to us and allows us to have a larger stage; the opportunity for team to meet the needs of members' development."

【DGM WXX】"Competing for a team is a process of rebirth."

【Staff member 8】"The open environment gives everyone the opportunity to compete, so that everyone has self-confidence, and the effort will always be rewarded."

【Staff member SGH】"The real competition is more about growth, popularization of knowledge and broadening of ideas."

【Staff member SGH】"If you can't change the environment, you have to change yourself. We left the starting line at the same starting point although we walked slowly."

【Staff member SGH】"The real competition is to motivate everyone to move forward. First of all, I would like to thank the open environment of the company for giving me an opportunity to show myself. I also especially thank my colleagues who have always encouraged me to improve... Competition is to use our changes to infect everyone in the team. One member, everyone wins."

【Supervisor YM】"SGH's success is the result of her own efforts, the company's platform is a stage for everyone's learning, and Debriefing and Competition Process are the results of the show. Hopefully role models like SGH and WK can inspire others."

【Supervisor PZW】"Although I failed in the competition, thank you for letting me reflect and let me grow!"

【Supervisor PZW】"I failed in the competition because there was neither data nor depth in the evaluation, which shows that the result of leading the team by relying on parental control in the past was a failure."

【Supervisor PZW】"Our team changed from 24 people to only 14 people now, in the final analysis, I should reflect on it."

【Supervisor PZW】"I can't just shout slogans or think that competition is formalism. Instead, I have to respect the rules and realize how to lead the team around and I want to train my subordinates because it's important for the team to cultivate talent resources."

【Team member 1】"At first, everyone may really regarded the competition as a formalism, but it was only after the result came out that they regretted it. Failure tests everyone like a raging fire, allowing everyone to reflect and take responsibility."

【Team member 2】"The failure of the executive competition is not a personal failure but our team's failure. But the failure is only temporary. Our slogan is not empty. In the future, we will do practical things and speak with attitude, and firmly believe that our department will get better and better! "

【Team member 3】"From the beginning of some confusion to the later perseverance, we have achieved reflection and reversal, and got rid of the management method of parental control, and truly achieved autonomy. I believe we will get better and better, come on!"

【Staff member 9】"Difficulties encountered at work are not necessarily a bad thing. It depends on how to solve the problem. It is really important to reflect the cohesion of the team at the critical moment to avoid recurrence or to deal with it correctly in the future."

【Supervisor WHL】"In my current experience, Only the ego first dissipates the team to dissipate."

【Supervisor DL】"Due to the lack of reverence for the rules and the lack of belief in the mechanism, the resurrection failed in the end. However, how can you see the rainbow without experiencing the wind and rain, and you will only be overcome with pain. During this period, the members changed from selfishness and laziness to self-dedication. Everyone really transformed from the inside out, showing team spirit."

【Staff member SXY】"Each part uses the weekend break to promote in the surrounding area in the form of independent points to attract a large number of tourists. Our hard work has paid off and it is the result of our concerted efforts."

【Supervisor PZW】"The successful implementation of the plan is the product of this independent struggle. It has gone through the process of consensus, self-approval, implementation, full-staff marketing, focusing on customer needs, self-responsibility, innovative proposals and final product generation."

Service innovation (Adding new service processes from the perspective of customer needs)

Product innovation (Developing new Entertainment Projects)

Marketing innovation (Expanding new target customer groups, members' independently adopt multi-way promotion strategies)
